# Assessing Coping Strategies and Outdoor Activities Among Older Adults During the COVID-19 Pandemic

**DOI:** 10.1093/geroni/igab046.2238

**Published:** 2021-12-17

**Authors:** Justine Sefcik, Martha Coates, Sarah Wetzel, Janvi Patel, Keyanna Bynum, K Linh Pham, Rose Ann DiMaria-Ghalili

**Affiliations:** 1 Drexel University, College of Nursing and Health Professions, Philadelphia, Pennsylvania, United States; 2 Drexel University, Bryn Mawr, Pennsylvania, United States; 3 Drexel University, Philadelphia, Pennsylvania, United States

## Abstract

Information is lacking on how older adults are coping during the pandemic. We explored coping strategies including outdoor activities among community-dwelling older adults (N = 115) 65 and older (mean age 76.45, 71.3% female). Using conventional content analysis, we analyzed responses to: 1) How are you coping with COVID-19? and 2) How often are you going outside during the pandemic and for what reasons? Most common activities are connecting with family and friends (some in person, others on the phone or virtually), reading, tv, game playing, and learning something new (e.g. webinars, online classes). The majority are going outside every day, with walking being the most common activity. Only a few are restricting their out of home activities to essential tasks (e.g. going to the doctors, pharmacy, getting groceries). Findings suggest that many older adults are engaging in positive coping activities. Assessing coping strategies can give insight into wellbeing.

